# Initial photophysical characterization of the proteorhodopsin optical proton sensor (PROPS)

**DOI:** 10.3389/fnins.2015.00315

**Published:** 2015-09-04

**Authors:** Jay L. Nadeau

**Affiliations:** Graduate Aerospace Laboratories, California Institute of TechnologyPasadena, CA, USA

**Keywords:** voltage-sensitive dyes, genetically encoded voltage sensor, proteorhodopsin, time-correlated single photon counting (TCSPC)

## Abstract

Fluorescence is not frequently used as a tool for investigating the photocycles of rhodopsins, largely because of the low quantum yield of the retinal chromophore. However, a new class of genetically encoded voltage sensors is based upon rhodopsins and their fluorescence. The first such sensor reported in the literature was the proteorhodopsin optical proton sensor (PROPS), which is capable of indicating membrane voltage changes in bacteria by means of changes in fluorescence. However, the properties of this fluorescence, such as its lifetime decay components and its origin in the protein photocycle, remain unknown. This paper reports steady-state and nanosecond time-resolved emission of this protein expressed in two strains of *Escherichia coli*, before and after membrane depolarization. The voltage-dependence of a particularly long lifetime component is established. Additional work to improve quantum yields and improve the general utility of PROPS is suggested.

## Introduction and background

Electrophysiology is the most sensitive technique available for measuring cell membrane potential, but patch-clamp recordings are labor intensive, can only be performed on a limited number of cells at a time, and are extremely difficult to perform on very small cells. One of the greatest technical challenges in neuroscience is to be able to perform optical recordings of real-time processes in large networks of coupled cells, the so-called “optical patch clamp.” To resolve a single action potential, a voltage-sensitive optical probe must have a potential resolution of ~100 mV or better, and a time resolution of milliseconds. Until recently, the best results were obtained using voltage-sensitive dyes, in particular a class of organic dyes called the amino-naphthyl-ethenyl-pyridinium (ANEP) dyes, such as di-4-ANEPPS and di-8-ANEPPS (Fluhler et al., 1985). While some groups have obtained action-potential data using these dyes, the technique is not widespread because of the specialized equipment needed and the low signal to noise in the best data (Tominaga et al., 2000; Tsutsui et al., 2001). Another, more sensitive dye-based approach involves detecting polarization changes in neurons by photo-induced electron transfer through a synthetic molecular wire to a dye (Miller et al., 2012). The speed of the electron transfer process makes this an ideal approach to monitoring fast voltage changes. However, dyes cannot be used in targeted cell populations or in whole animals. Genetically encoded alternatives have been sought for several decades, with significant breakthroughs appearing within the past several years.

### Approaches to genetically encoded voltage indicators (GEVIs)

GEVIs have been thoroughly reviewed in several articles (Baker et al., 2008; Frommer et al., 2009; Akemann et al., 2010; Mutoh et al., 2012; Ohba et al., 2013). The general approach to creating a genetically encoded voltage sensor is to fuse a fluorescent reporter, usually from the family of green fluorescent protein (GFP), with a voltage-sensing domain (VSD) in such a way that the conformational changes of the sensor with voltage result in a change in the fluorescence of the reporter. However, because of the robustness of GFP fluorescence, slowness of fluorescent response to perturbations in the molecule, and lack of expression of membrane protein-tagged GFPs (Baker et al., 2007), changing emission substantially in this fashion has proven to be a difficult task.

**Figure 1:**
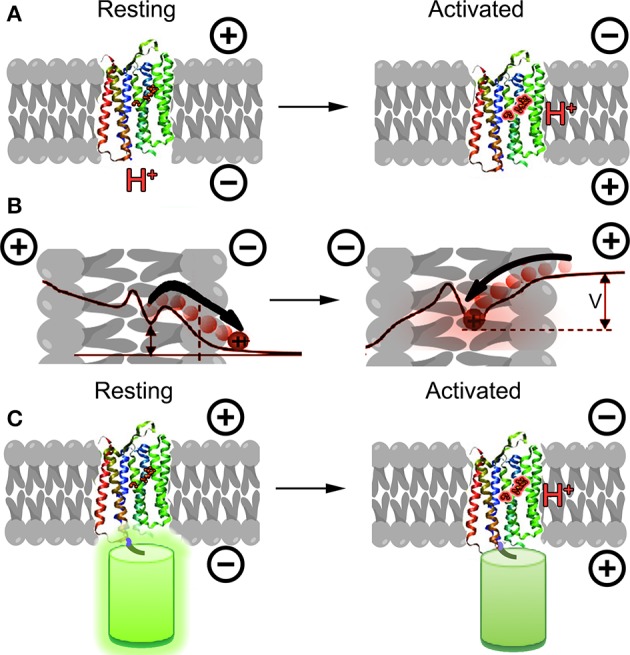
**Opsins as GEVIs. (A)** Principle of opsin-based sensors. The retinal chromophore within the protein becomes fluorescent when protonated as a result of membrane potential depolarization. **(B)** Detailed mechanism of PROPS voltage sensing (concept from Kralj et al., 2011). When membrane potential is negative, protons move away from the Schiff base, causing the chromophore to become less fluorescent. As the membrane depolarizes, protons move toward the Schiff base and increase the quantum yield of the chromophore. The ratio of protonated to deprontonated species depends upon the voltage drop *V* between the Schiff base and the cytoplasm; in general *V* < *V*_*mem*_. **(C)** A new concept for opsin-based GEVIs fuses fluorescent proteins to the opsin and uses differences in FRET efficiency between the fluorescent protein and the retinal for sensing.

An entirely new alternative approach emerged in 2011 based upon microbial opsins rather than the GFP family. These proteins transduce light into cellular signals, including changes in membrane potential; the concept behind engineering them into voltage sensors was to reverse this relationship, transducing changes in membrane potential into changes in fluorescence emission. The first demonstration of this principle was made using a proteorhodopsin-based optical proton sensor (PROPS) from green light-absorbing bacteria (Figure 1A) (Kralj et al., 2011). The principle of PROPS is that a Schiff base is located on the proteorhodopsin inside the membrane. When Vm < 0, protons move from the base to the cytoplasm, causing the protein to become non-fluorescent. When Vm > 0, protons move from the cytoplasm onto the base, causing an increase in fluorescence. The ratio of protonated to deprotonated Schiff bases depends upon the voltage drop between the membrane protein and the cytoplasm (Figure 1B).

This proof of principle has been significantly developed over the past few years. PROPS does not target well to plasma membranes of eukaryotic cells, so the group developed a similar sensor based upon archaerhodopsin-3 (Arch), an optogenetic control tool (Kralj et al., 2012; Maclaurin et al., 2013; Hou et al., 2014; Venkatachalam et al., 2014). Most recently, the opsin principle has been used to develop electrochromic FRET-based voltage sensors (Figure 1C) (Gong et al., 2014; Zou et al., 2014). These rely upon FRET between the opsin and attached fluorescent protein, and required significant optimization of the protein choice and length of linker.

### Bacterial ion channels and neuroscience

Although it cannot be used in mammalian cells, PROPS remains interesting as both a proof of principle and as a bacterial sensor. In order to study electrogenic properties of bacterial membrane proteins, the proteins are usually cloned and expressed in *Xenopus* oocytes, which removes the downstream effects seen in the native cells (Schmies et al., 2001). The role of membrane potential in prokaryotic cell signaling is well known, but not fully understood (Szmelcman and Adler, 1976; Margolin and Eisenbach, 1984; Ordal, 1985; Tisa et al., 1993). When elucidated, the mechanisms used by bacteria to regulate membrane potential may help shed light on evolution of memory, olfaction, and other complex functions (Eisenbach, 1982; Eisenbach et al., 1983a,b; Goulbourne and Greenberg, 1983; Vladimirov and Sourjik, 2009; Lyon, 2015). Bacterial ion channels are also often good models for the function of mammalian ion channels, and their relationship to membrane potential may perhaps provide new approaches to drug screening. For example, the bacterium *Arcobacter butzleri* was recently found to have a voltage-gated Na^+^ channel, whose selectivity filter is profoundly different from that seen in mammalian cells (Payandeh et al., 2011). The significance of this remains unknown. Despite limited sequence homology, bacterial sodium channels and transporters share binding sites with mammalian homologs, and often respond to the same ligands (Henry et al., 2007; Bagnéris et al., 2014). The development of bacterial-based optical screening techniques for drugs affecting the sodium channel, as well as other channels and transporters, would have immense application in neuroscience (Chakrabarti et al., 2013; Bagnéris et al., 2014).

In this paper we perform steady-state and time-resolved spectroscopy of PROPS expressed in two bacterial strains. The dependence of emission brightness, spectrum, and lifetime were studied as a function of wavelength and power of excitation. Fits to 1–3 Gaussian distributions were necessary to describe the lifetime decays, consistent with the chromophore being embedded within a protein. Depolarization of the cells with CCCP or exposure to violet light led to greater population of the longer-lifetime state, consistent with changes in steady-state intensity observed during microscopy. Voltage dependence of this fluorescent state was observed. Based upon these results, a preliminary model for fluorescence in PROPS is suggested, with ideas for future work.

## Materials and methods

### Strains and expression

The *E. coli* strains containing PROPS were a gift of Adam Cohen, Harvard University. They were prepared as reported previously: *E. coli* was grown to early-log phase (OD600 = 0.3–0.4) in Lysogeny Broth (LB) at 33°C. Arabinose was added as an inducer along with 5 μM all-trans retinal; further growth was conducted in the dark. The cells were harvested 3.5 h after induction and washed with minimal medium (1x M9 salts, 0.4% glucose, pH 7), then resuspended in minimal medium. Cultures were stored at 4°C for up to 1 week before use. Two different strains of *E. coli* were used: BW25113 (referred to here as “BW”) (Δ(araD-araB)567, ΔlacZ4787(::rrnB-3), lambda-, rph-1, Δ(rhaDrhaB) 568, hsdR514; and UT5600 (the “UT” strain) (F- ara-14 leuB6 secA6 lacY1 proC14 tsx-67 Δ(ompT-fepC)266 entA403 trpE38 rfbD1 rpsL109 xyl-5 mtl-1 thi-1). Concentrations of arabinose used for induction were 0.02% w/v (BW strain) or 0.2% (UT strain), and protein expressed was gauged by the color of the pellet.

### Steady-state spectroscopy and TCSPC

Steady-state spectra were recorded on a Fluorolog-3 (Jobin Yvon) spectrometer. Measurements were made with and without the addition of 50 μg/mL carbonyl cyanide *m*-chlorophenyl hydrazone (CCCP; Sigma-Aldrich). Photoluminescence decays from bulk samples were obtained by the time-correlated single photon counting (TCSPC) technique. Eight hundred nano meter laser pulses (~70 fs) out of a Coherent RegA 9050 Ti/sapphire regenerative amplifier operating at 250 kHz repetition rate were used to pump an OPA (Coherent 9450) which produced tunable visible light with an average power of ~30 mW. The beam was focused into the sample with a focal spot diameter of ~0.25 mm. The excitation power delivered to the sample was set at 3 mW (“High Power”) or 40 μW (“Low Power”). For violet light exposure, full power at 400 nm was used, providing ~50 W/cm^2^; exposure was performed before spectroscopy because only one illumination wavelength was possible at a time. The luminescence was collected with a 3.5 cm focal length lens placed perpendicular to the excitation beam and the collimated luminescence focused into a monochromator with a 10 cm focal length lens. The monochromator was a CVI CMSP112 double spectrograph with a 1/8 m total path length in negative dispersive mode with a pair of 600 groove/mm gratings (overall *f* number 3.9). The slit widths were 2.4 mm and based on a monochromator dispersion of 14 nm/mm, provided 10 nm resolution. A Hamamatsu RU3809 microchannel-plate photomultiplier was mounted on the monochromator exit slit. A Becker and Hickl SPC-630 photon counting board was used to record the time-resolved emission. The reference signal was provided by a portion of the excitation beam sent to a fast photodiode. To ensure good statistics, count rates were held at < 1% of the laser repetition rate to avoid pulse pile up. Typical acquisition times were 10 min for a single scan. The instrument response function (IRF) was determined from scatter off a solution of dilute coffee creamer. The full width at half-maximum of the IRF was 37 ps.

### Curve fitting

Data analysis was performed using FluoFit 4.0 (PicoQuant, Berlin). Goodness of fit was assessed by χ^2^-values and by examination of residuals; χ^2^-values < 1.1 and a random distribution of residuals were required for a fit to be considered accurate. A sum of exponentials, up to 4 terms, was insufficient to describe the decays, as was a stretched exponential (up to 3 terms). The best fit was obtained to a sum of Gaussian distributions, which is appropriate for a collection fluorophores within inhomogeneous environments such as proteins, with the mathematics and physics developed by Prendergast et al. (Alcala et al., 1987a,b,c; Togashi and Ryder, 2006). This model is described by the equations.

(1) $$\begin{array}{l} I(t)=\int_{-\infty }^{t}IRF(t^{\prime })\int_{-\infty }^{\infty }\rho (\tau )\text{exp}\left(-\frac{t-t^{\prime }}{\tau }\right)d\tau dt^{\prime }\\ \rho (\tau )=\sum_{i\;=\;1}^{n}\frac{A_{i}}{\sigma_{i}\sqrt{2\pi }}\text{exp}\left[-\frac{1}{2}{\left(\frac{\tau -\tau_{i}}{\sigma_{i}}\right)}^{2}\right]\\ \;\sigma_{i}=\frac{\Delta_{FWHMi}}{\sqrt{\text{8ln2}}}, \end{array}$$

where *n* = 1 − 3 for our samples. Both amplitude-weighted average lifetimes:

$$\begin{array}{l} \langle \tau \rangle =\underset{i}{\sum }\frac{A_{i}\tau_{i}}{A_{i}} \end{array}$$

and intensity-weighted average lifetimes:

$$\begin{array}{l} \langle \tau \rangle =\underset{i}{\sum }\frac{A_{i}{\tau_{i}}^{2}}{A_{i}\tau_{i}} \end{array}$$

were calculated. The intensity average corresponds to the amount of time the fluorophore spends in the excited state. The amplitude average is the lifetime a fluorophore would have if it had the same steady-state fluorescence as the fluorophore with several lifetimes (Sillen and Engelborghs, 1998).

## Results

**Figure 2:**
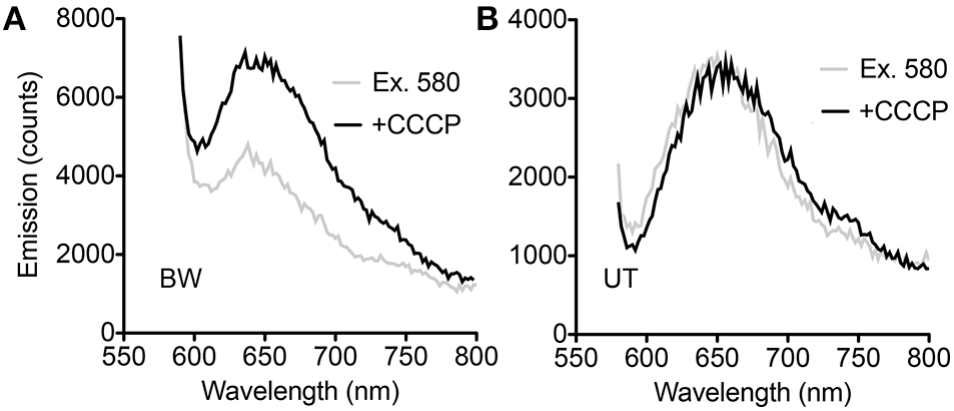
**Steady-state spectra. (A)** Difference between spectra with and without CCCP with excitation at 580 nm in the BW strain. **(B)** Difference between spectra with and without CCCP with excitation at 580 nm in the UT strain.

All spectroscopy was performed on PROPS expressed in *E. coli* cells. Steady-state spectroscopy showed a fluorescence emission peak at ~660 nm in both strains after induction; there was no measurable emission without induction. In the BW strain, an increase in emission with CCCP was seen across the emission peak when samples were excited at 580 nm (Figure 2A). In the UT strain, smaller differences were noted at wavelengths both bluer and redder than the emission peak (Figure 2B).

**Figure 3:**
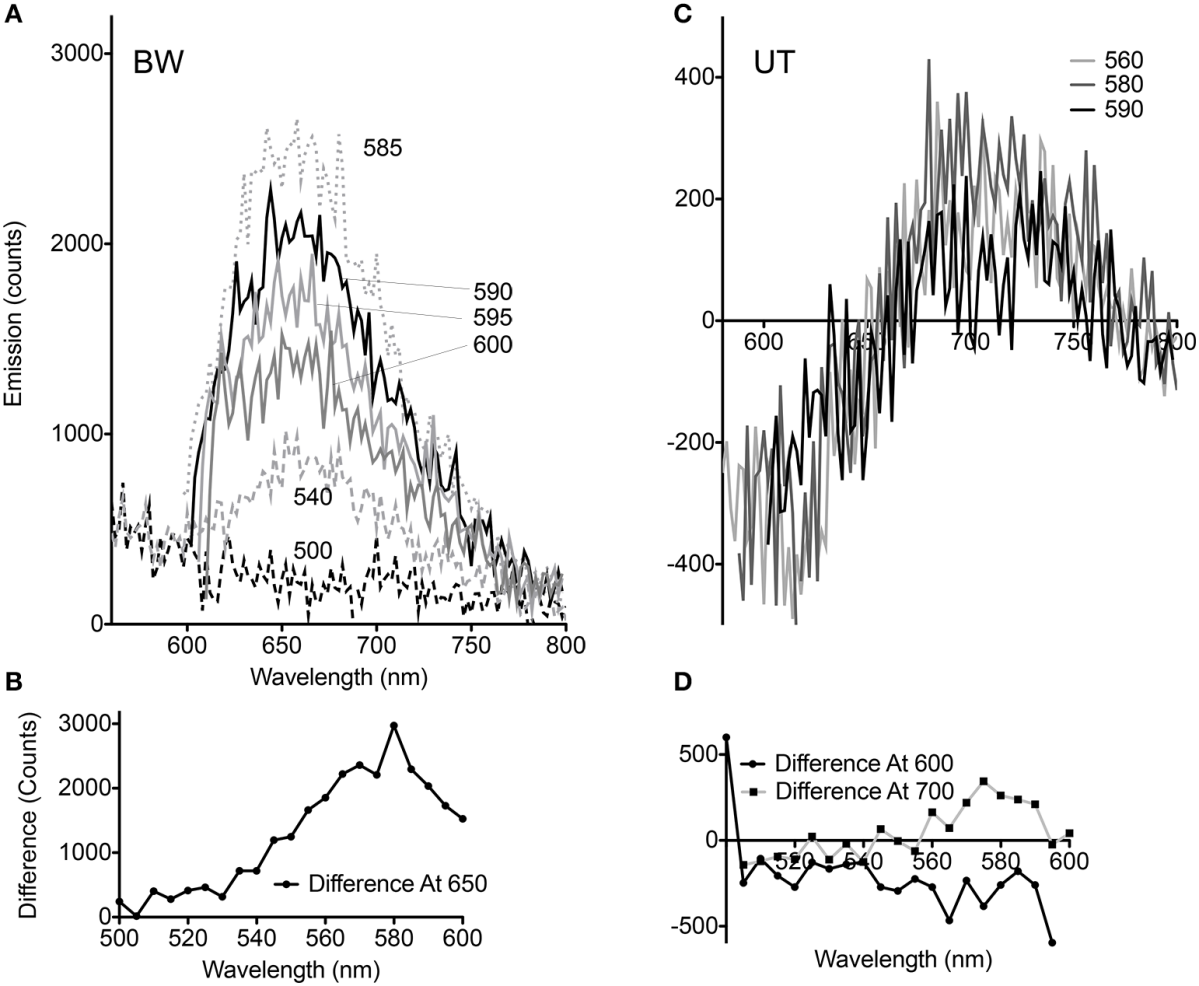
**Dependence of voltage-sensitivity upon wavelength of excitation. (A)** Difference spectra (CCCP-no CCCP) for multiple excitation wavelengths in the BW strain. The different excitation wavelengths are indicated by numbers next to the curves. **(B)** Difference at 650 nm emission vs. excitation wavelength in the BW strain. **(C)** Difference spectra (CCCP-no CCCP) for multiple excitation wavelengths in the UT strain. **(D)** Difference at 600 and 700 nm emission vs. excitation wavelength in the UT strain.

Although the pellets of both strains appeared equally pink, comparison of the spectral changes seen with CCCP at different excitation wavelengths revealed significant differences for the BW strain. At excitation wavelengths from 510 to 530 nm, essentially no change was seen. Between 535 and 580 nm, the difference with CCCP grew in a roughly linear fashion, then declined again in approximately a mirror image of the increase. Excitation wavelengths >600 nm were not used so that the entire spectral peak could be captured (Figures 3A,B). The UT strain was significantly different. Less dependence upon excitation wavelength was seen in the difference spectra, and rather than reflect a simple enhancement or quenching, the spectral changes showed a negative and a positive peak. Overall changes were significantly smaller than with the BW strain. Although the absolute value of the emission was approximately half as strong in the UT strain (peak ~7000 counts for BW vs. ~3500 counts for UT), the differences with CCCP were almost 10-fold lower in this strain than in the BW strain (difference of ~300 counts with CCCP for UT vs. nearly 3000 for BW) (Figures 3C,D).

**Figure 4:**
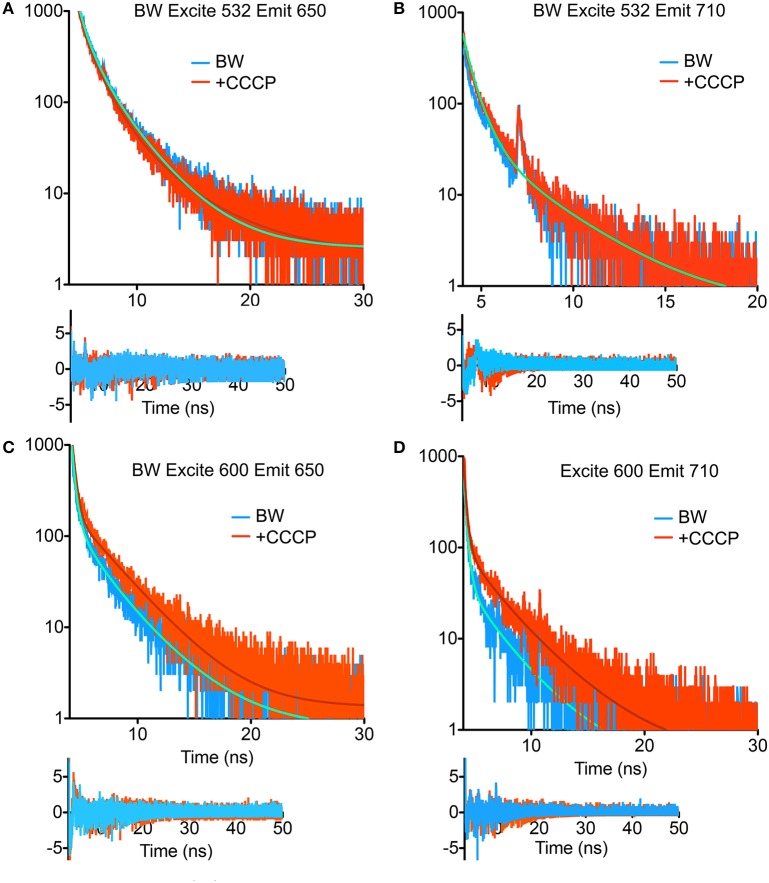
**Fluorescence lifetime decays of the PROPS-expressing BW strain**. Fits are to the parameters shown in Tables 1, 2 with residuals shown beneath the curves. **(A)** Excitation at 532 nm, emission at 650 nm. **(B)** Excitation at 532 nm, emission at 710 nm. The fits for the two curves overlap. **(C)** Excitation at 600 nm, emission at 650 nm. **(D)** Excitation at 600 nm, emission at 710 nm.

TCSPC was then performed at 532 and 600 nm excitation with the two strains, beginning with the BW strain at low power (40 μW). With excitation at 532 nm and emission at 650 nm, three terms were required in Equation (1) to obtain a good fit. Although there was a longer-lifetime component apparent in the samples after the addition of CCCP, both the intensity-weighted and amplitude-weighted average lifetimes were indistinguishable in the two cases (Figure 4A, Table 1). Emission at 710 nm could be fit to a single Gaussian-distributed exponential without CCCP. Addition of CCCP again caused the appearance of a longer lifetime component, but without change in mean lifetime (Figure 4B, Table 1). Excitation at high power (3 mW) caused the complete disappearance of the longer-lifetime component, with a significant reduction in mean lifetime (Table 1).

**Table 1:** **TCSPC fit parameters for the BW strain with excitation at 532 nm**.

**Fit parameters**	**BW Excite 532 Emit 650**	**BW Excite 532 Emit 650 + CCCP**	**BW Excite 532 Emit 710**	**BW Excite 532 Emit 710 + CCCP**	**BW Excite 532 Emit 710 High Power**	**BW Excite 532 Emit 710 +CCCP High**
A_1_ [Cnts]	697	152	584	41.4	14,149	11,651
τ1 [ns]	0.945	3.11	0.677	3.10	0.396	0.396
ΔFWHM 1 [ns]	0.001	4.59	1.10	0.001	0.134	0.316
A_2_ [Cnts]	10,604	7829	x	1879	186,400	47,690
τ2 [ns]	0.280	0.225	x	0.307	0.036	0.052
ΔFWHM 2 [ns]	0.456	0.361	x	0.681	0.026	0.016
A_3_ [Cnts]	3694	8510	x	x	6690	4470
τ3 [ns]	0.001	0.019	x	x	0.0014	0.094
ΔFWHM 3 [ns]	3.76	2.09	x	x	3.58	3.44
τAv.1 (intensity weighted)	1.08	1.13	0.970	0.970	0.629	0.751
τAv.2 (amplitude weighted)	0.496	0.458	0.738	0.442	0.080	0.161
Fraction intensity 1	10.9	8.94	100	17.2	24.9	46.5
Fraction intensity 2	50.1	33.5	0	82.8	34.3	25.1
Fraction intensity 3	39.0	57.6	0	0	40.8	28.4
Fraction amplitude 1	4.65	0.92	100	2.16	6.83	18.3
Fraction amplitude 2	70.7	47.47	0	97.8	89.9	74.7
Fraction amplitude 3	24.6	51.60	0	0	3.23	7.01

**Figure 5:**
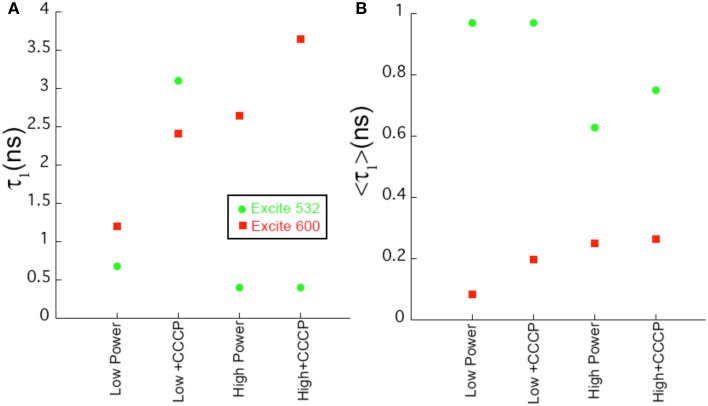
**Comparison of fit parameters. (A)** Length of the longest lifetime component as a function of sample conditions for selected samples. **(B)** Value of the intensity-weighted average lifetime as a function of sample conditions. Uncertainties in the fits are < 2%.

In contrast, at 600 nm excitation voltage dependence of the lifetime decays could be observed. At 650 nm emission, the longest-lifetime component shifted from 1.2 to 2.4 ns with the addition of CCCP. In addition, the fractional intensity of the longer component increased. Both the intensity-weighted and amplitude-weighted lifetimes were approximately doubled with CCCP addition at 650 and 710 nm emission (Figures 4C,D, Table 2). Pre-exposure to violet light (400 nm, 50 W/cm^2^) had a nearly identical effect. CCCP plus violet light led to a further increase in the longest lifetime and a slight increase in its fractional intensity. High power excitation further increased the magnitude of the long lifetime (Table 2). Figure 5 illustrates the long-lifetime component and mean intensity-weighted lifetime as a function of selected test conditions. It can be readily seen from Figure 5A that a lifetime component >2 ns occurred with CCCP or high-power excitation with 600 nm light. While a long component was seen with 532 nm excitation in the presence of CCCP at low excitation power, high excitation power at 532 suppressed this component. From Figure 5B, it can be appreciated that the mean lifetimes were voltage-dependent only with 600 nm excitation.

**Table 2:** **TCSPC fit parameters for the BW strain with excitation at 600 nm**.

**Fit parameters**	**BW Emit 650**	**BW Emit 650 +Violet**	**BW Emit 650 +CCCP**	**BW Emit 650 CCCP +Violet**	**BW Emit 710**	**BW Emit 710 +CCCP**	**BW Emit 710 +CCCP +Violet**	**BW Emit 650 High**	**BW Emit 650 +CCCP High**
A_1_ [Cnts]	340	170	259	214	60	141	141	172	137
τ1 [ns]	1.20	2.65	2.41	3.03	2.06	2.25	2.62	2.65	3.65
ΔFWHM 1 [ns]	3.71	2.08	0.95	1.14	2.91	3.26	1.19	0.31	0.001
A_2_ [Cnts]	32,390	5886	12,657	6648	7357	10,369	11,632	6293	247,600
τ2 [ns]	0.061	0.246	0.203	0.287	0.081	0.120	0.213	0.267	0.027
ΔFWHM 2 [ns]	0.308	0.001	0.107	0.001	0.261	0.196	0.116	0.015	0.002
A_3_ [Cnts]	1,387,000	173,700	348,600	211,900	x	x	3,175,000	162,300	32,060
τ3 [ns]	0.018	0.035	0.025	0.036	x	x	0.015	0.032	0.002
ΔFWHM 3 [ns]	0.022	0.001	0.019	0.017	x	x	0.020	0.022	0.413
τAv.1 (intensity weighted)	0.083	0.238	0.197	0.279	0.567	0.752	0.049	0.251	0.263
τAv.2 (amplitude weighted)	0.0205	0.0442	0.0328	0.0465	0.143	0.167	0.0169	0.044	0.036
Frac intensity 1	1.7	5.66	5.27	6.35	15.04	20.18	0.72	6.21	5.24
Frac intensity 2	10.2	18.23	21.62	18.73	84.96	79.82	4.80	22.9	70.75
Frac intensity 3	88.1	76.11	73.12	74.92	0	0	94.48	70.9	24.01
Frac amplitude 1	0.02	0.09	0.07	0.10	0.81	1.34	0.00	0.10	0.05
Frac amplitude 2	2.28	3.27	3.50	3.04	99.19	98.66	0.36	3.73	88.49
Frac amplitude 3	97.69	96.63	96.43	96.86	0	0	99.63	96.2	11.46

The results for the UT strain were qualitatively similar, though the magnitude of the changes was smaller than with the BW strain, consistent with what was observed in the steady-state spectra. Only emission at 710 nm was recorded because this was a maximum in the difference spectra. Excitation at 600 nm led to a small increase in mean lifetime. High-power excitation reduced lifetime. Interestingly, a small decrease in mean lifetime was seen with CCCP addition at 532 nm excitation (Table 3).

**Table 3:** **TCSPC fit parameters for the UT strain with excitation at 532 and 600 nm**.

**Fit parameters**	**UT Excite 532 Emit 710 Low**	**UT Excite 532 Emit 710 + CCCP Low**	**UT Excite 532 Emit 710 High**	**UT Excite 532 Emit 710 High +CCCP**	**UT Excite 600 Emit 710**	**UT Excite 600 Emit 710 +CCCP**	**UT Excite 600 Emit 710 +Violet**
A_1_ [Cnts]	147	101	332	328	76.59	51.25	27.78
τ1 [ns]	2.02	2.31	0.144	1.01	0.90	1.12	1.18
ΔFWHM 1 [ns]	0.73	0.79	4.71	3.43	1.27	0.62	0.39
A_2_ [Cnts]	19,482	1607	26,780	12,332	2376	2057	1001
τ2 [ns]	0.132	0.362	0.112	0.213	0.186	0.120	0.195
ΔFWHM 2 [ns]	0.154	0.001	0.204	0.001	0.001	0.002	0.002
A_3_ [Cnts]	x	32,300	x	1,084,000	35,050	18,500	7261
τ3 [ns]	x	0.083	x	0.025	0.028	0.034	0.034
ΔFWHM 3 [ns]	x	0.092	x	0.011	0.001	0.001	0.001
τAv.1 (intensity weighted)	0.362	0.294	0.377	0.072	0.130	0.156	0.184
τAv.2 (amplitude weighted)	0.150	0.11	0.139	0.028	0.040	0.052	0.052
Frac intensity 1	10.4	6.6	8.54	1.3	4.68	5.41	7.08
Frac intensity 2	89.6	16.6	91.46	8.7	29.6	37.7	41.9
Frac intensity 3	0	76.8	0	90.0	65.7	56.9	51.0
Frac amplitude 1	0.75	0.3	1.2	0.03	0.20	0.25	0.34
Frac amplitude 2	99.25	4.7	98.8	1.12	6.34	9.98	12.1
Frac amplitude 3	0	95	0	98.85	93.5	89.8	87.6

## Discussion

The results obtained here are consistent with the original work on PROPS, which reported increased red fluorescence in PROPS-expressing *E. coli* upon depolarization with CCCP or exposure to violet light (Kralj et al., 2011). Our results suggest that there are multiple red-fluorescent species in heterogeneous environments within the protein, but that most of them have much shorter lifetimes than the species responsible for the voltage-sensitive emission, which has a lifetime of ~2.5–3 ns. Only a fraction of the molecules probed in these experiments showed this slower lifetime, suggesting that improvements in PROPS yield and voltage sensitivity could be obtained through exact identification and mutation of this state to yield greater stability or ease of excitation.

The differences seen between the two tested strains might have been due to a two-fold difference in expression levels, with the BW strain expressing more highly. However, it may also reflect differences in membrane potential due to strain variations or differences in the phase of the cell cycle in which the cells were harvested, which can affect membrane potential (Bot and Prodan, 2010). Control for the phase of growth should be performed in future studies of PROPS in *E. coli* if quantitative comparisons are desired. It is also possible that PROPS is less well trafficked in the UT strain. Poorly trafficked proteins will appear as inclusion bodies in cells, so would be readily identified upon high-resolution optical microscopy if the cells were used for optical recording. A comparison of growth rates and viable cells might also show differences in toxicity of the protein to the different strains.

**Figure 6:**
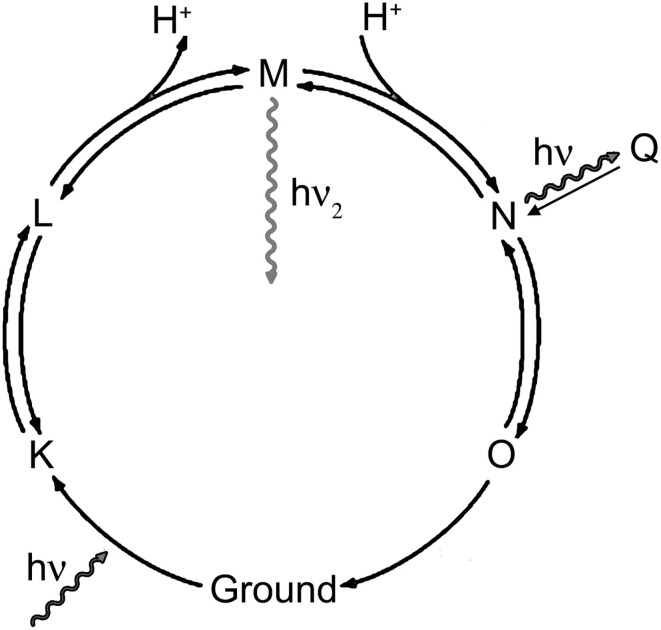
**Rhodopsin photocycle**. The exact lifetimes and absorbance spectra for each state vary from protein to protein, but in general hν represents green light and hν_2_ represents violet light.

The association of the emissive state with stages in the proteorhodopsin photocycle cannot be done precisely from these data, but a simplified model may be suggested based upon the observations in analogy with what is known about other proteins. Proteorhodopsin has a photocycle similar to that of bacteriorhodpsin, where a proton is moved across the membrane by means of a series of conformational changes (Figure 6). Retinal begins as all-*trans* in the ground state (G), and the Schiff base is protonated. Photoisomerization of retinal to the *cis* results from visible excitation and results in the L state. The Schiff base is then deprotonated to the extracellular side (M1), then becomes accessible to the cytoplasmic side (M2). It is reprotonated to form the N state, and the retinal returns to the *trans* state (O). The O state then returns to ground. Fluorescence can result from on-pathway states or from off-pathway states that are created by the light excitation. Key observations from the current study are: (1) the longer-lifetime state is excited efficiently at 600 nm, but not at 532 nm; (2) high laser power (3 mW) prevents the observation of this state with 532 nm emission, but enhances it with 600 nm emission; (3) depolarization and violet light exposure both enhance the fraction of molecules in this state, and the effects of the two are additive.

The observed CCCP dependence of the fluorescence might be suspected to result from changes in pH of the cells, rather than necessarily from depolarization. The original study deconvolved these effects by co-expressing pHluorin with PROPS. When cells were treated with CCCP, intracellular pH became equal to extracellular pH, leading in most cases to a change in fluorescence of pHluorin. However, PROPS fluorescence increased sharply regardless of medium pH—even when the internal and external pH were identical—suggesting that its fluorescence changes were due to membrane potential rather than pH (Kralj et al., 2011). In the current work, with external pH ~7, the fluorescence changes in PROPS are due to both membrane potential and pH.

The photophysics of bacteriorhodopsin (Cao et al., 1993; Kamiya et al., 1997, 1999) and of an opsin-based GEVI, Arch (Maclaurin et al., 2013), have been investigated in great detail. The fluorescence of Arch was initially believed to result from the ground state, but instead was found to be the result of a state formed from the N state by exposure to yellow light, called the Q state. A similar Q state has also been observed in bR due to the sequential absorption of 3 photons (Ohtani et al., 1992, 1995). While green light is sufficient to create Q from N, orange light is required for excitation of Q.

It is likely that the fluorescence here results from Q. Increased laser power increases PROPS quantum yield with orange light excitation but not with green. Since Q is a 3-photon process, its formation should increase at higher laser power, and it is excited with orange rather than green light. It is unlikely that the voltage-sensitive PROPS fluorescence arises from the O state or the ground state, since in these states the Schiff base is extracellular, and the voltage-sensitive state it is cytoplasmic. Further studies using simultaneous red and violet light exposure, and perhaps transient absorption, will be needed to elucidate the precise identity of the emitting states. The utility of the present work is in identifying the lifetime of the fluorescent state, which is comparable to that seen with the GFP family (Pepperkok et al., 1999). These results illustrate the utility of nanosecond-scale fluorescence measurements, and suggest experiments to screen for mutants that show better quantum yields than the currently available constructs. Complementing steady-state brightness results, time-resolved measurements distinguish between mutations that increase lifetime and those which increase the fraction of emission from the longer-lifetime state. Such measurements may help identify mutations that create novel states with substantially increased lifetime, as has been done with cyan fluorescent protein (Goedhart et al., 2010).

These results also suggest approaches to the use of GEVIs in fluorescence lifetime imaging microscopy (FLIM). FLIM is a valuable technique for quantitative fluorescence microscopy because it does not depend upon fluorophore concentration. The existing construct has too low of a quantum yield to be seen with commercial FLIM (data not shown), but custom microscopes and/or improved mutants may make this a valuable approach to voltage sensing. The current work shows that the appearance of the 2–3 ns lifetime is a signal for protonation of the Schiff base, an ideal lifetime range for FLIM, as the signal is able to decay between laser pulses. The small magnitude of the voltage-dependent change seen in the current studies is almost certainly due to the presence of many inhomogeneous proteins in these bulk samples, so it is likely that changes of single molecules will be resolvable on a system that is set up for imaging PROPS.

## Conclusion

The fluorescence emission of PROPS results from a variety of states, of which the voltage dependent state has a lifetime of ~2–3 ns and probably corresponds to the orange-excited Q state. Fluorescence lifetime measurements can provide insight into the photophysics of GEVIs, which can lead to improved sensors and to the use of such sensors in applications such as FLIM.

### Conflict of interest statement

The author declares that the research was conducted in the absence of any commercial or financial relationships that could be construed as a potential conflict of interest.

## References

[B1] Akemann W. Mutoh H. Perron A. Rossier J. Knöepfel T. (2010). Imaging brain electric signals with genetically targeted voltage-sensitive fluorescent proteins. Nat. Methods 7, U643–U664. 10.1038/nmeth.1479 20622860

[B2] Alcala J. R. Gratton E. Prendergast F. G. (1987a). Fluorescence lifetime distributions in proteins. Biophys. J. 51, 597–604. 10.1016/S0006-3495(87)83384-2 3580486 PMC1329931

[B3] Alcala J. R. Gratton E. Prendergast F. G. (1987b). Interpretation of fluorescence decays in proteins using continuous lifetime distributions. Biophys. J. 51, 925–936. 10.1016/S0006-3495(87)83420-3 3607213 PMC1330026

[B4] Alcala J. R. Gratton E. Prendergast F. G. (1987c). Resolvability of fluorescence lifetime distributions using phase fluorometry. Biophys. J. 51, 587–596. 10.1016/S0006-3495(87)83383-0 3580485 PMC1329930

[B5] Bagnéris C. Decaen P. G. Naylor C. E. Pryde D. C. Nobeli I. Clapham D. E.. (2014). Prokaryotic NavMs channel as a structural and functional model for eukaryotic sodium channel antagonism. Proc. Natl. Acad. Sci. U.S.A. 111, 8428–8433. 10.1073/pnas.1406855111 24850863 PMC4060673

[B6] Baker B. J. Lee H. Pieribone V. A. Cohen L. B. Isacoff E. Y. Knopfel T.. (2007). Three fluorescent protein voltage sensors exhibit low plasma membrane expression in mammalian cells. J. Neurosci. Methods 161, 32–38. 10.1016/j.jneumeth.2006.10.005 17126911

[B7] Baker B. J. Mutoh H. Dimitrov D. Akemann W. Perron A. Iwamoto Y.. (2008). Genetically encoded fluorescent sensors of membrane potential. Brain Cell Biol. 36, 53–67. 10.1007/s11068-008-9026-7 18679801 PMC2775812

[B8] Bot C. T. Prodan C. (2010). Quantifying the membrane potential during *E. coli* growth stages. Biophys. Chem. 146, 133–137. 10.1016/j.bpc.2009.11.005 20031298

[B9] Cao Y. Brown L. S. Needleman R. Lanyi J. K. (1993). Relationship of proton uptake on the cytoplasmic surface and reisomerization of the retinal in the bacteriorhodopsin photocycle—an attempt to understand the complex kinetics of the pH changes and the N and O intermediates. Biochemistry 32, 10239–10248. 10.1021/bi00089a046 8399152

[B10] Chakrabarti N. Ing C. Payandeh J. Zheng N. Catterall W. A. Pomés R. (2013). Catalysis of Na^+^ permeation in the bacterial sodium channel Na(V)Ab. Proc. Natl. Acad. Sci. U.S.A. 110, 11331–11336. 10.1073/pnas.1309452110 23803856 PMC3710854

[B11] Eisenbach M. (1982). Changes in membrane-potential of *Escherichia-coli* in response to temporal gradients of chemicals. Biochemistry 21, 6818–6825. 10.1021/bi00269a030 6760894

[B12] Eisenbach M. Raz T. Ciobotariu A. (1983a). A process related to membrane-potential involved in bacterial chemotaxis to galactose. Biochemistry 22, 3293–3298. 10.1021/bi00282a039 6349685

[B13] Eisenbach M. Zimmerman J. R. Ciobotariu A. Fischler H. Korenstein R. (1983b). Electric-field effects on bacterial motility and chemotaxis. Bioelectrochem. Bioenerget. 10, 499–510. 10.1016/0302-4598(83)80077-4

[B14] Fluhler E. Burnham V. G. Loew L. M. (1985). Spectra, membrane binding, and potentiometric responses of new charge shift probes. Biochemistry 24, 5749–5755. 10.1021/bi00342a010 4084490

[B15] Frommer W. B. Davidson M. W. Campbell R. E. (2009). Genetically encoded biosensors based on engineered fluorescent proteins. Chem. Soc. Rev. 38, 2833–2841. 10.1039/b907749a 19771330 PMC3000468

[B16] Goedhart J. van Weeren L. Hink M. A. Vischer N. O. Jalink K. Gadella T. W. Jr. (2010). Bright cyan fluorescent protein variants identified by fluorescence lifetime screening. Nat. Methods 7, 137–139. 10.1038/nmeth.1415 20081836

[B17] Gong Y. Wagner M. J. Zhong Li J. Schnitzer M. J. (2014). Imaging neural spiking in brain tissue using FRET-opsin protein voltage sensors. Nat. Commun. 5, 3674. 10.1038/ncomms4674 24755708 PMC4247277

[B18] Goulbourne E. A. Greenberg E. P. (1983). A voltage clamp inhibits chemotaxis of spirochaeta-aurantia. J. Bacteriol. 153, 916–920. 6822479 10.1128/jb.153.2.916-920.1983 PMC221714

[B19] Henry L. K. Meiler J. Blakely R. D. (2007). Bound to be different: neurotransmitter transporters meet their bacterial cousins. Mol. Interv. 7, 306–309. 10.1124/mi.7.6.4 18199851

[B20] Hou J. H. Venkatachalam V. Cohen A. E. (2014). Temporal dynamics of microbial rhodopsin fluorescence reports absolute membrane voltage. Biophys. J. 106, 639–648. 10.1016/j.bpj.2013.11.4493 24507604 PMC3945107

[B21] Kamiya N. Ishikawa M. Kasahara K. Kaneko M. Yamamoto N. Ohtani H. (1997). Picosecond fluorescence spectroscopy of the purple membrane of Halobacterium halobium in alkaline suspension. Chem. Phys. Lett. 265, 595–599. 10.1016/S0009-2614(97)01509-1

[B22] Kamiya N. Ohtani H. Sekikawa T. Kobayashi T. (1999). Sub-picosecond fluorescence spectroscopy of the M intermediate in the photocycle of bacteriorhodopsin by using up-conversion fluorometry. Chem. Phys. Lett. 305, 15–20. 10.1016/S0009-2614(99)00342-5

[B23] Kralj J. M. Douglass A. D. Hochbaum D. R. Maclaurin D. Cohen A. E. (2012). Optical recording of action potentials in mammalian neurons using a microbial rhodopsin. Nat. Methods 9, 90–95. 10.1038/nmeth.1782 22120467 PMC3248630

[B24] Kralj J. M. Hochbaum D. R. Douglass A. D. Cohen A. E. (2011). Electrical spiking in *Escherichia coli* probed with a fluorescent voltage-indicating protein. Science 333, 345–348. 10.1126/science.1204763 21764748

[B25] Lyon P. (2015). The cognitive cell: bacterial behavior reconsidered. Front. Microbiol. 6:264. 10.3389/fmicb.2015.00264 25926819 PMC4396460

[B26] Maclaurin D. Venkatachalam V. Lee H. Cohen A. E. (2013). Mechanism of voltage-sensitive fluorescence in a microbial rhodopsin. Proc. Natl. Acad. Sci. U.S.A. 110, 5939–5944. 10.1073/pnas.1215595110 23530193 PMC3625274

[B27] Margolin Y. Eisenbach M. (1984). Voltage clamp effects on bacterial chemotaxis. J. Bacteriol. 159, 605–610. 6430873 10.1128/jb.159.2.605-610.1984 PMC215686

[B28] Miller E. W. Lin J. Y. Frady E. P. Steinbach P. A. Kristan W. B. Jr. Tsien R. Y. (2012). Optically monitoring voltage in neurons by photo-induced electron transfer through molecular wires. Proc. Natl. Acad. Sci. U.S.A. 109, 2114–2119. 10.1073/pnas.1120694109 22308458 PMC3277584

[B29] Mutoh H. Akemann W. Knöpfel T. (2012). Genetically engineered fluorescent voltage reporters. ACS Chem. Neurosci. 3, 585–592. 10.1021/cn300041b 22896802 PMC3419450

[B30] Ohba Y. Fujioka Y. Nakada S. Tsuda M. (2013). Fluorescent protein-based biosensors and their clinical applications, in Fluorescence-Based Biosensors: From Concepts to Applications, ed Morris M. C. (Waltham, MA: AcademicPress; Elsevier), 313–348. 10.1016/B978-0-12-386932-6.00008-9 23244794

[B31] Ohtani H. Itoh H. Shinmura T. (1992). Time-resolved fluorometry of purple membrane of Halobacterium-halobium—O(640) and an O-like red-shifted intermediate-Q. FEBS Lett. 305, 6–8. 10.1016/0014-5793(92)80643-U 1633860

[B32] Ohtani H. Tsukamoto Y. Sakoda Y. Hamaguchi H. (1995). Fluorescence-spectra of bacteriorhodopsin and the intermediate-O and intermediate-Q at room-temperature. FEBS Lett. 359, 65–68. 10.1016/0014-5793(94)01440-C 7851532

[B33] Ordal G. W. (1985). Bacterial chemotaxis—biochemistry of behavior in a single cell. CRC Crit. Rev. Microbiol. 12, 95–130. 10.3109/10408418509104426 2992881

[B34] Payandeh J. Scheuer T. Zheng N. Catterall W. A. (2011). The crystal structure of a voltage-gated sodium channel. Nature 475, 353–358. 10.1038/nature10238 21743477 PMC3266868

[B35] Pepperkok R. Squire A. Geley S. Bastiaens P. I. (1999). Simultaneous detection of multiple green fluorescent proteins in live cells by fluorescence lifetime imaging microscopy. Curr. Biol. 9, 269–272. 10.1016/S0960-9822(99)80117-1 10074454

[B36] Schmies G. Engelhard M. Wood P. G. Nagel G. Bamberg E. (2001). Electrophysiological characterization of specific interactions between bacterial sensory rhodopsins and their transducers. Proc. Natl. Acad. Sci. U.S.A. 98, 1555–1559. 10.1073/pnas.98.4.1555 11171989 PMC29295

[B37] Sillen A. Engelborghs Y. (1998). The correct use of “Average” fluorescence parameters. Photochem. Photobiol. 67, 475–486. 24185058

[B38] Szmelcman S. Adler J. (1976). Change in membrane-potential during bacterial chemotaxis. Proc. Natl. Acad. Sci. U.S.A. 73, 4387–4391. 10.1073/pnas.73.12.4387 794876 PMC431468

[B39] Tisa L. S. Olivera B. M. Adler J. (1993). Inhibition of *Escherichia-coli* chemotaxis by omega-conotoxin, a calcium-ion channel blocker. J. Bacteriol. 175, 1235–1238. 8444785 10.1128/jb.175.5.1235-1238.1993 PMC193206

[B40] Togashi D. M. Ryder A. G. (2006). Time-resolved fluorescence studies on bovine serum albumin denaturation process. J. Fluoresc. 16, 153–160. 10.1007/s10895-005-0029-9 16382334

[B41] Tominaga T. Tominaga Y. Yamada H. Matsumoto G. Ichikawa M. (2000). Quantification of optical signals with electrophysiological signals in neural activities of Di-4-ANEPPS stained rat hippocampal slices. J. Neurosci. Methods 102, 11–23. 10.1016/S0165-0270(00)00270-3 11000407

[B42] Tsutsui H. Wolf A. M. Knöpfel T. Oka Y. (2001). Imaging postsynaptic activities of teleost thalamic neurons at single cell resolution using a voltage-sensitive dye. Neurosci. Lett. 312, 17–20. 10.1016/S0304-3940(01)02177-2 11578835

[B43] Venkatachalam V. Brinks D. Maclaurin D. Hochbaum D. Kralj J. Cohen A. E. (2014). Flash memory: photochemical imprinting of neuronal action potentials onto a microbial rhodopsin. J. Am. Chem. Soc. 136, 2529–2537. 10.1021/ja411338t 24428326 PMC3985752

[B44] Vladimirov N. Sourjik V. (2009). Chemotaxis: how bacteria use memory. Biol. Chem. 390, 1097–1104. 10.1515/BC.2009.130 19747082

[B45] Zou P. Zhao Y. Douglass A. D. Hochbaum D. R. Brinks D. Werley C. A.. (2014). Bright and fast multicoloured voltage reporters via electrochromic FRET. Nat. Commun. 5, 4625. 10.1038/ncomms5625 25118186 PMC4134104

